# Effect of Nudges on Downloads of COVID-19 Exposure Notification Apps: A Randomized Clinical Trial

**DOI:** 10.1001/jamanetworkopen.2021.40839

**Published:** 2021-12-23

**Authors:** Marissa A. Sharif, Erica Dixon, Elizabeth F. Bair, Carolina Garzon, Laura Gibson, Kristin Linn, Kevin Volpp

**Affiliations:** 1 Marketing Department, Wharton School of the University of Pennsylvania, Philadelphia; 2 Center for Health Incentives and Behavioral Economics (CHIBE) at the University of Pennsylvania School of Medicine, Philadelphia; 3 Department of Medical Ethics and Health Policy, Perelman School of Medicine, University of Pennsylvania, Philadelphia; 4 Center for Health Care Innovation, Penn Medicine, Philadelphia, Pennsylvania; 5 Department of Biostatistics, Epidemiology, and Informatics at the University of Pennsylvania School of Medicine, Philadelphia

## Abstract

This randomized clinical trial examines the effect of digital contact tracing using smartphone app nudges to increase downloads of Pennsylvania’s COVID Alert PA app.

## Introduction

Digital contact tracing smartphone applications (apps) can mitigate the spread of COVID-19 through exposure notification.^1,2,3,4^ However, their success requires widespread use. We examined the effectiveness of low-cost behavioral interventions (ie, nudges) in increasing downloads of Pennsylvania’s COVID Alert PA app. Specifically, we explored the effectiveness of 2 nudges^5,6^ on 39 937 individuals, one nudge displaying a descriptive social norm (vs not) and another framing the benefit of downloading for others (vs self).

## Method

In this randomized clinical trial, 39 937 Pennsylvania members of Independence Blue Cross were randomly assigned to receive 1 email in a 2 (focus on self vs focus on others) × 2 (social norm vs no social norm) design (Table 1). In the focus-on-self condition, the message stated the benefits of the app for participants as follows: “It can help you determine where and when to get testing, and how to get care if you need it.” In the focus-on-others condition, the message stated the benefit to others as follows: “It can help you reduce your risk of unknowingly spreading the virus to your friends, family, and larger community.” In the social-norm condition, the message stated, “Over 650 000 Pennsylvanians have already downloaded the app!” This statement was absent in the no-social-norm condition. At the bottom of each email, a link directed participants to a site to download the app (eMethods in Supplement 1). This study was exempt from University of Pennsylvania Institutional Review Board approval and received a waiver for informed consent because the study involved minimal risk to the participants. The full trial protocol appears in Supplement 2. This study followed the Consolidated Standards of Reporting Trials (CONSORT) reporting guideline.

**Table 1.** Baseline Demographics for Each Condition

Characteristic	Participants by group, No. (%)				
	Overall (N = 39 937)	Other benefit		Self-benefit	
		Without social norm (n = 9993)	With social norm (n = 9982)	Without social norm (n = 9979)	With social norm (n = 9983)
Age, mean (SD), y	46.5 (13.2)	46.3 (13.2)	46.5 (13.2)	46.7 (13.0)	46.4 (13.3)
Sex					
Male	16 624 (41.6)	4202 (42.0)	4166 (41.7)	4112 (41.2)	4144 (41.5)
Female	23 313 (58.4)	5791 (58.0)	5816 (58.3)	5867 (58.8)	5839 (58.5)
Action					
Email opened	16 903 (42.3)	4214 (42.2)	4211 (42.2)	4246 (42.6)	4232 (42.4)
Download link clicked^a^	1832 (10.8)	744 (17.7)	355 (8.4)	380 (9.0)	353 (8.3)

^a^ Percentages are among participants who opened the email.

We conducted 1 logistic regression estimating our primary outcome, clicks on the download link contingent on opening the email (42.3% opened), from 2 dummy variables representing conditions and a similar regression with the interaction term in the model. A 2-sided, α = .05 was considered statistically significant. Both were intent-to-treat analyses using Stata, version 15.1 (StataCorp LLC).

## Results

Of 39 937 participants, the mean (SD) age was 46.5 (13.2) years and 41.6% were male. Findings show participants were significantly more likely to click on the link when a social norm was absent (vs present) (no social norm = 13.3% vs social norm = 8.4%; unadjusted OR, 0.60; 95% CI, 0.54-0.66; *P* < .001) and when the message focused on the benefits to others (vs self) (focus on others = 13.0% vs focus on self = 8.7%; unadjusted OR, 0.63; 95% CI, 0.57-0.69; *P* < .001).

The main effects were qualified by a significant 2 (focus on self vs focus on others) × 2 (social norm vs no social norm) interaction (unadjusted OR, 2.16; 95% CI, 1.76-2.64; *P* < .001). When the message focused on the benefit to others, including a social norm significantly decreased clicks on the link (focus on others, no social norm = 17.7% vs focus on others, social norm = 10.9%; unadjusted OR, 0.43; 95% CI, 0.38-0.49; *P* < .001; Table 2). When the message focused on the benefit to self, including a social norm did not have a significant effect (focus on self, no social norm = 11.4% vs focus on self, social norm = 10.7%; unadjusted OR, 0.93; 95% CI, 0.80-1.08; *P* = .32). The most effective nudge focused on the benefits for others without a social norm, leading to a 6.0% or greater increase in clicks relative to other conditions.

**Table 2.** Link Clicks Estimated via Logistic Regression^a^

Variable	Unadjusted model (n = 16 903)		Adjusted model (n = 16 903)	
	OR (95% CI)	*P* value	OR (95% CI)	*P* value
Focus on self	0.46 (0.40-0.52)	<.001	0.46 (0.40-0.52)	<.001
Social norm	0.43 (0.38-0.49)	<.001	0.43 (0.37-0.49)	<.001
Focus on self × social norm interaction	2.16 (1.76-2.64)	<.001	2.17 (1.77-2.66)	<.001
Age	NA	NA	1.50 (1.36-1.66)	<.001
Sex	NA	NA	1.02 (1.02-1.02)	<.001
Intercept of regression model	0.21 (0.20-0.23)	<.001	0.07 (0.06-0.09)	<.001

Abbreviation: NA, not applicable.

^a^ Logistic regression results predicting link clicks (1 = click; 0 = no click) from a dummy variable representing the social norm (vs no social norm) condition (1 = social norm; 0 = no social norm) and a dummy variable representing the focus on self (vs focus on others condition) (1 = focus on self; 0 = focus on others), and a variable representing the interaction.

## Discussion

The findings of this randomized clinical trial reveal the effect of social norm and self (vs other) nudges on downloads of a contact tracing app. One limitation of this study was its reliance on an indirect measure of downloads, that is, clicks to a website to download.

Future research should explore the mechanism and whether the effect generalizes to other locations/apps. One possibility is that when focusing on the benefit to others, downloading the app is perceived as a group goal, which may be motivating with no social norm. However, when a norm signals many already contributed to the goal (without a specific target), people may believe their individual action is less needed. Overall, this research suggests that costless nudges can help reduce the spread of harmful viruses by increasing downloads of contact tracing apps, an important and urgent issue.

## Notes

Supplement 1. **eMethods.** Supplemental Methods

Supplement 2.
Trial Protocol

Supplement 3.
Data Sharing Statement

## References

[1] Ferretti L, Wymant C, Kendall M, . Quantifying SARS-CoV-2 transmission suggests epidemic control with digital contact tracing. Science. 2020;368(6491):eabb6936. doi:10.1126/science.abb6936 32234805 PMC7164555

[2] Aleta A, Martín-Corral D, Pastore Y Piontti A, . Modelling the impact of testing, contact tracing and household quarantine on second waves of COVID-19. Nat Hum Behav. 2020;4(9):964-971. doi:10.1038/s41562-020-0931-9 32759985 PMC7641501

[3] Rodríguez P, Graña S, Alvarez-León EE, ; RadarCovidPilot Group. A population-based controlled experiment assessing the epidemiological impact of digital contact tracing. Nat Commun. 2021;12(1):587. doi:10.1038/s41467-020-20817-6 33500407 PMC7838392

[4] Abueg M, Hinch R, Wu N, . Modeling the effect of exposure notification and non-pharmaceutical interventions on COVID-19 transmission in Washington state. NPJ Digit Med. 2021;4(1):49. doi:10.1038/s41746-021-00422-7 33712693 PMC7955120

[5] Chung A, Rimal RN. Social norms: a review. Review of Communication Research. 2016;4:1-28. doi:10.12840/issn.2255-4165.2016.04.01.008

[6] Kelly BJ, Hornik RC. Effects of framing health messages in terms of benefits to loved ones or others: an experimental study. Health Commun. 2016;31(10):1284-1290. doi:10.1080/10410236.2015.1062976 26940483

