# Supplementary material for: Effect of Nudges on Downloads of COVID-19 Exposure Notification Apps: A Randomized Clinical Trial
Source: JAMA Netw Open. 2021 Dec 23;4(12):e2140839. doi: 10.1001/jamanetworkopen.2021.40839 (PMC8703239; doi:10.1001/jamanetworkopen.2021.40839)
Supplement: Supplement 1. — eMethods. Supplemental Methods [file jamanetwopen-e2140839-s001.pdf]

## Supplemental Online Content

Sharif MA, Dixon E, Bair EF, et al. Effect of nudges on downloads of COVID-19 exposure notification apps: a randomized clinical trial. *JAMA Netw Open*. 2021;4(12):e2140839. doi:10.1001/jamanetworkopen.2021.40839

**eMethods.** Supplemental Methods

This supplemental material has been provided by the authors to give readers additional information about their work.

© 2021 Sharif MA et al. *JAMA Network Open*.

**eMethods. Supplemental Methods**

Eligible participants were active beneficiaries of a large insurer living in Pennsylvania. Participants who did not subscribe to offers and promotions emails were excluded, as were Medigap beneficiaries. Within this sample, 41.6% were male sex and 58.4% were female sex (Age Mean = 46.5, SD =13.2, 41.6% Male).

**Sample size**

We did not do an a priori sample size calculation because we knew there would be no marginal cost to adding extra participants, and planned on asking the insurer to provide us with the largest sample size they could accommodate. The insurer was willing to send emails to approximately 40,000 participants, as their mailings cap at 10,000 per mailing and we had 4 conditions.

**Flow diagram**

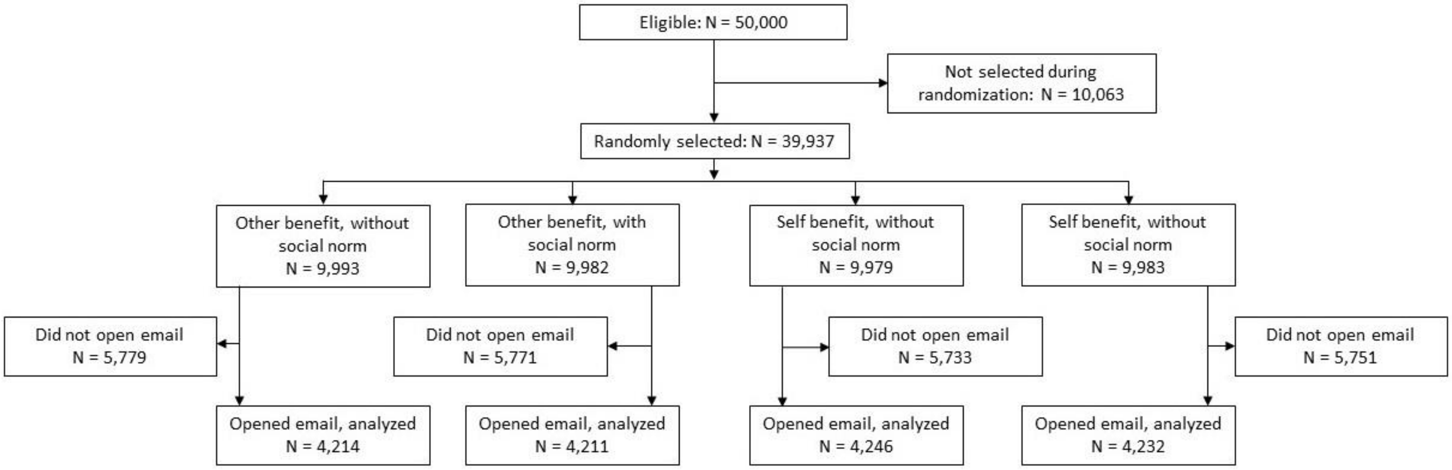


**Randomization and blinding**

The large insurer implemented computer generated the simple randomization sequences, assigned participants to interventions, and sent out the email interventions. From the eligible sample of 50,000, potential participants were randomly assigned with 1:1:1:1 allocation to one of the four treatments. Beneficiaries were unaware they were participating in a study, and were thus blinded to their treatment assignment.

**Intervention**

The emails for each arm are included below:

1. **Focus on Others & No Social Norm**

   **Subject Line:** COVID-19 exposure? Get notified!
   **Preview Text:** Reduce the risk of spreading the virus to friends, family, and community.
   **E-mail Body:**

© 2021 Sharif MA et al. *JAMA Network Open*.

COVID Alert PA is the official Exposure Notification App from the Pennsylvania Department of Health. Join Independence Blue Cross in the fight against COVID-19 and download COVID Alert PA today!

Getting an alert through the app lets you know if you have had a potential exposure to someone who tested positive for COVID-19.

**It can help you reduce your risk of unknowingly spreading the virus to your friends, family, and larger community. Over 650,000 Pennsylvanians have already downloaded the app!***

The app uses Bluetooth to sense when one person is in close contact with another person with the app. If someone tests positive for COVID-19, and they decide to upload the information to the health department, people who have the app and who have been in contact with them will be alerted.

If you are interested in downloading the app, please download COVID Alert PA.
We appreciate you joining our efforts. Together we can beat COVID-19!

2. **Focus On Others & Social Norm**

**Subject Line:** COVID-19 exposure? Get notified!
**Preview Text:** Over 650,000 Pennsylvanians have already downloaded the app!
**E-mail Body:**

COVID Alert PA is the official Exposure Notification App from the Pennsylvania Department of Health. Join Independence Blue Cross in the fight against COVID-19 and download COVID Alert PA today!

Getting an alert through the app lets you know if you have had a potential exposure to someone who tested positive for COVID-19.

**It can help you reduce your risk of unknowingly spreading the virus to your friends, family, and larger community. Over 650,000 Pennsylvanians have already downloaded the app!***

The app uses Bluetooth to sense when one person is in close contact with another person with the app. If someone tests positive for COVID-19, and they decide to upload the information to the health department, people who have the app and who have been in contact with them will be alerted.

If you are interested in downloading the app, please download COVID Alert PA.
We appreciate you joining our efforts. Together we can beat COVID-19!

3. **Focus on Self & No Social Norm**

**Subject Line**: COVID-19 exposure? Get notified!
**Preview Text:** Determine where and when to get testing, and how to get care if you need it.
**E-mail Body:**

COVID Alert PA is the official Exposure Notification App from the Pennsylvania Department of Health. Join Independence Blue Cross in the fight against COVID-19 and download COVID Alert PA today!

© 2021 Sharif MA et al. *JAMA Network Open*.

Getting an alert through the app lets you know if you have had a potential exposure to someone who tested positive for COVID-19.

**It can help you determine where and when to get testing, and how to get care if you need it.***

The app uses Bluetooth to sense when one person is in close contact with another person with the app. If someone tests positive for COVID-19, and they decide to upload the information to the health department, people who have the app and who have been in contact with them will be alerted.

If you are interested in downloading the app, please download COVID Alert PA.
We appreciate you joining our efforts. Together we can beat COVID-19!

4. **Focus on Self & Social Norm**

**Subject Line:** COVID-19 exposure? Get notified!
**Preview Text:** Over 650,000 Pennsylvanians have already downloaded the app!
**E-mail Body**:

COVID Alert PA is the official Exposure Notification App from the Pennsylvania Department of Health. Join Independence Blue Cross in the fight against COVID-19 and download COVID Alert PA today!

Getting an alert through the app lets you know if you have had a potential exposure to someone who tested positive for COVID-19.

**It can help you determine where and when to get testing, and how to get care if you need it. Over 650,000 Pennsylvanians have already downloaded the app!***

The app uses Bluetooth to sense when one person is in close contact with another person with the app. If someone tests positive for COVID-19, and they decide to upload the information to the health department, people who have the app and who have been in contact with them will be alerted.

If you are interested in downloading the app, please download COVID Alert PA.
We appreciate you joining our efforts. Together we can beat COVID-19!

The initial emails were sent out to all participants on December 11, 2020. A follow-up email was sent to all participants who had not opened the first email on December 18, 2020, and follow-up ended on January 28, 2021.

*Indicates the sentence is bolded for emphasis to show differences between messages, but was *not* bold in e-mails that were sent.

As our intervention was an email encouraging participants to download the COVID-Alert PA app, there was no potential harm to participants. Participants could only benefit by helping to reduce the spread of COVID-19.

Our study was pre-registered at aspredicted.org: https://aspredicted.org/NEK_LJJ

© 2021 Sharif MA et al. *JAMA Network Open*.
