# Supplementary material for: Effect of Nudges on Downloads of COVID-19 Exposure Notification Apps: A Randomized Clinical Trial
Source: JAMA Netw Open. 2021 Dec 23;4(12):e2140839. doi: 10.1001/jamanetworkopen.2021.40839 (PMC8703239; doi:10.1001/jamanetworkopen.2021.40839)
Supplement: Supplement 2. — Trial Protocol [file jamanetwopen-e2140839-s002.pdf]

ASPREDICTED

Wharton CREDIBILITY LAB
UNIVERSITY of PENNSYLVANIA

Self Benefit vs. Other Benefit X Social Norm (#54102)

Created: 12/11/2020 11:11 AM (PT)
Public: 11/18/2021 10:53 AM (PT)

Author(s)
Marissa A. Sharif (University of Pennsylvania/The Wharton School) - masharif@wharton.upenn.edu
Elizabeth Bair (University of Pennsylvania) - efbair@pennmedicine.upenn.edu
Erica Dixon (University of Pennsylvania) - erica.dixon@pennmedicine.upenn.edu
Kevin Volpp (University of Pennsylvania) - volpp70@wharton.upenn.edu
Kristin Linn (University of Pennsylvania) - klinn@pennmedicine.upenn.edu

1) Have any data been collected for this study already?
No, no data have been collected for this study yet.

2) What's the main question being asked or hypothesis being tested in this study?
How does framing the COVID-Alert PA app in terms of self-benefit vs. other-benefit, as well including information about the number of people who have downloaded the app thus far (or not), influence the decision to download the COVID-Alert PA app?

3) Describe the key dependent variable(s) specifying how they will be measured.
Our main dependent variable is the choice to click on a link to download the COVID-Alert PA app or not.

4) How many and which conditions will participants be assigned to?
Participants will be randomly assigned to one of four conditions in a 2 (Self-benefit vs. Other-Benefit) x 2 (Social Norms Present vs. Social Norms absent) design.

All participants will receive an email that says:
COVID Alert PA is the official Exposure Notification App from the Pennsylvania Department of Health. Join Penn Medicine in the fight against COVID-19 and download COVID Alert PA today!
---We will manipulate the content here---

The app uses Bluetooth to sense when one person is in close contact with another person with the app. If someone tests positive for COVID-19, and they decide to upload the information to the health department, people who have the app and who have been in contact with them will be alerted.

If you are interested in downloading the app, please click COVID Alert PA.
We appreciate you joining our efforts. Together we can beat COVID-19!

In the Other-Benefit condition, it will say: It can help you reduce your risk of unknowingly spreading the virus to your friends, family, and larger community.

In the Self-Benefit condition, it will say: It can help you determine where and when to get testing, and how to get care if you need it.

In the Social Norms Present condition, it will say: Over 600,000 Pennsylvanians have already downloaded the app! [This number may change slightly if the number of people who downloaded the app changed from the time of the preregistration and the time of the launch]

In the Social Norms Absent condition, it will not say the statement about social norms above.

Participants who do not open the initial email will receive a follow-up email.

5) Specify exactly which analyses you will conduct to examine the main question/hypothesis.
We will conduct a logistic regression predicting choice to click on the link or not from a dummy variable representing the Self-Benefit (vs. Other-Benefit) condition, the Social Norms Present (vs. Social Norms Absent) condition, and a variable representing the interaction.

We will examine if there is a 2 (Self-Benefit vs. Other-Benefit) x 2 (Social Norms Present vs. Social Norms absent) interaction. If there is not a significant interaction, we will examine if there is a main effect of Self-Benefit (vs. Other-Benefit) and Social Norms Present (vs. Social Norms Absent).

Depending on the availability of race, age, and gender demographic variables, we will also conduct the analyses above controlling for these variables.

6) Describe exactly how outliers will be defined and handled, and your precise rule(s) for excluding observations.
We will only include participants in our analysis that have opened the sent email.

7) How many observations will be collected or what will determine sample size? No need to justify decision, but be precise about exactly how the number will be determined.

The emails will be sent to approximately 40,000 people (10,000 per condition).

8) Anything else you would like to pre-register? (e.g., secondary analyses, variables collected for exploratory purposes, unusual analyses planned?)

NA
