# Supplementary material for: Effect of Nudges on Downloads of COVID-19 Exposure Notification Apps: A Randomized Clinical Trial
Source: JAMA Netw Open. 2021 Dec 23;4(12):e2140839. doi: 10.1001/jamanetworkopen.2021.40839 (PMC8703239; doi:10.1001/jamanetworkopen.2021.40839)
Supplement: Supplement 3. — Data Sharing Statement [file jamanetwopen-e2140839-s003.pdf]

Data Sharing Statement

Sharif. Effect of Nudges on Downloads of COVID-19 Exposure Notification Apps. *JAMA Netw Open*. Published December 23, 2021. doi:10.1001/jamanetworkopen.2021.40839

Data
Data available: No
